# Biomechanical Evaluation of Patient-Specific Titanium Fixation Systems for Segmental Le Fort I Osteotomy: A Finite Element Analysis

**DOI:** 10.3390/jfb17090454

**Published:** 2026-09-07

**Authors:** Neculai Onică, Elena-Raluca Baciu, Cezara Andreea Onică, Florin-Emilian Țurcanu, Geta-Mirela Ispas, Vlad Nicolae Arsenoaia, Loredana Golovcencu, Alice Murariu

**Affiliations:** 1 Grigore T. Popa University of Medicine and Pharmacy Iasi, 700115 Iasi, Romania; onica_neculai@d.umfiasi.ro (N.O.); cezara-andreea.onica@umfiasi.ro (C.A.O.); loredana.golovcencu@umfiasi.ro (L.G.); alice.murariu@umfiasi.ro (A.M.); 2 VEA MFG L.T.D., Bratuleni, 707307 Iasi, Romania; 3 Technical University “Gheorghe Asachi” of Iasi, 700050 Iasi, Romania; florin-emilian.turcanu@academic.tuiasi.ro; 4 “Ion Ionescu de la Brad” Iasi University of Life Sciences, 700490 Iasi, Romania; mirela.ispas@iuls.ro (G.-M.I.); nicolae.arsenoaia@iuls.ro (V.N.A.)

**Keywords:** patient-specific plate, Le Fort I osteotomy, Ti–6Al–4V, locking screw interface, finite element analysis, craniofacial biomechanics

## Abstract

Patient-specific Ti–6Al–4V fixation systems are increasingly used to stabilise the maxilla following segmental Le Fort I osteotomy; however, the biomechanical responses of complete locking and non-locking patient-specific fixation configurations remain insufficiently characterised. In this study, two patient-specific plate designs (a standard design and a reduced, minimally invasive design) were analysed by linear-elastic finite element analysis, each with locking and non-locking screws. Because they were developed for different indications and evaluated in distinct patient-specific anatomical models, between-model comparisons were considered descriptive. Vertical forces of 97.6, 206.9, and 257.0 N were applied bilaterally to the incisor and premolar regions, and the equivalent von Mises stress and total displacement were evaluated in the bone, plates, and screws. All results scaled linearly with load. Within the standard model, the locking configuration exhibited slightly greater bone, plate, and screw displacement than the corresponding non-locking configuration. Within the reduced model, it exhibited lower maximum bone, plate, and screw displacement by 16.4%, 19.2%, and 16.3%, respectively. Solver-reported local stress maxima were concentrated at the screw-head/plate-hole transition; because these isolated peaks were contact- and mesh-sensitive, they were not used to quantify relative screw loading or predict yielding. The modelled locking and non-locking fixation configurations therefore exhibited different, model-dependent displacement patterns. Further controlled simulations and experimental validation are required to determine the clinical relevance of these findings.

## 1. Introduction

Orthognathic surgery has been an essential component of craniomaxillofacial surgery for more than a century and remains the gold-standard treatment for the correction of dentofacial deformities. Modern maxillary and mandibular osteotomies facilitate the three-dimensional (3D) repositioning of the maxillomandibular complex, resulting in improved occlusal function, enhanced facial aesthetics, greater skeletal stability, and, in selected cases, enlargement of the upper airway with a subsequent improvement in airway function [1,2,3]. In recent years, the integration of digital technologies has fundamentally transformed orthognathic surgical workflows, enabling highly accurate virtual planning and improved transfer of the preoperative plan to the operating room [4,5,6].

Maxillary osteotomy using the Le Fort I approach is the standard surgical procedure for the correction of complex dentofacial deformities affecting the midface [7]. Rigid internal fixation with titanium plates and screws remains the clinical standard for stabilising the mobilised maxillary segments [8,9]. Nevertheless, postoperative relapse may occur because of unfavourable loading, insufficient construct stability or inadequate force transmission across the osteotomy sites [10]. Its magnitude varies according to the direction and extent of maxillary movement, segmentation pattern and fixation strategy [11,12]. Although comparisons of two- and four-plate fixation have not demonstrated a consistent overall difference in skeletal stability, reduced fixation may be less predictable following larger advancements [13,14]. Therefore, the optimal fixation configuration remains incompletely defined [15].

Advances in virtual surgical planning, CAD/CAM technologies and additive manufacturing have enabled the development of patient-specific titanium fixation systems that conform to the individual maxillary anatomy [16]. These systems can improve the transfer accuracy of the planned maxillary position [17] and can be adapted to the nasomaxillary and zygomaticomaxillary buttresses to support load transmission [18,19]. Their biomechanical performance, however, depends on the complete fixation configuration, including plate geometry, screw distribution and screw–plate engagement.

Ti–6Al–4V is widely used for craniomaxillofacial fixation because of its favourable strength-to-weight ratio, corrosion resistance and biocompatibility. In additively manufactured devices, its mechanical response is also influenced by build orientation, thermal history, residual stresses, surface condition and post-processing [20].

Finite element analysis (FEA) has become a valuable computational tool for investigating the mechanical behaviour of biological tissues and fixation systems under simulated physiological conditions. By enabling detailed assessment of stress distribution, displacement patterns, and structural stability, FEA provides important insights into the biomechanical performance of fixation systems without the limitations associated with in vivo experimentation and has consequently become a well-established methodology across traumatology, orthognathic surgery, reconstructive surgery, and implantology in the oral and maxillofacial field [21,22].

Previous biomechanical and clinical investigations have shown that plate number and position, screw distribution and plate geometry influence load transfer and the stability of the osteotomised maxilla [18,19,23]. Patient-specific fixation systems have also been compared with conventional miniplates in segmental Le Fort I osteotomy [24], while locking and non-locking patient-specific fixation has been investigated in mandibular reconstruction [25]. Nevertheless, evidence regarding the biomechanical response of complete locking and non-locking patient-specific configurations within the same maxillary anatomy and under multiple functional loading levels remains limited. Addressing this knowledge gap is important for the biomechanical optimisation of patient-specific fixation systems and the selection of fixation configurations that ensure adequate stability while minimising excessive local stress concentrations.

Accordingly, this study aimed to compare the biomechanical responses of complete locking and non-locking fixation configurations within each of two patient-specific models for segmental Le Fort I osteotomy under identical loading and boundary conditions. Equivalent von Mises stress localisation and maximum total displacement were evaluated in the maxillary bone, fixation plates, and screws under low, intermediate, and high functional loads of 97.6, 206.9, and 257.0 N. Within-model comparisons constituted the primary basis for interpretation, whereas comparisons between the two patient-specific anatomical models were considered descriptive.

## 2. Materials and Methods

### 2.1. Patient Selection and Three-Dimensional Model Reconstruction

This computational finite element study was conducted to evaluate the biomechanical performance of patient-specific fixation systems for segmental Le Fort I osteotomy using finite element analysis. Preoperative cone-beam computed tomography (CBCT) scans from two adult patients with skeletal Class II and Class III dentofacial deformities requiring orthognathic surgery were used. The scans were acquired using a DentriMax unit (HDX Will, Seoul, Republic of Korea), operated at 90 kV and 10 mA, with a field of view of 18 × 16.5 cm and a focal spot size of 0.5 mm. The CBCT datasets were used to reconstruct two patient-specific three-dimensional cranio-maxillofacial models. The two patient-specific anatomical models represented distinct clinical indications for the standard and reduced fixation designs. Each design was therefore evaluated within its intended anatomical setting rather than by applying both plate geometries to the same patient-specific model. Because plate geometry and topology, screw number and distribution, skeletal class, planned surgical movement, and local buttress anatomy differed simultaneously between the models, between-model comparisons were considered descriptive and cannot isolate the effect or establish the superiority of either plate design.

The study was conducted in accordance with the ethical principles of the Declaration of Helsinki and was approved by the Research Ethics Committee of the Grigore T. Popa University of Medicine and Pharmacy, Iași, Romania (Approval No.566/ Approval date:18 March 2025).

The CBCT datasets used for three-dimensional reconstruction had an in-plane pixel spacing of 0.4 × 0.4 mm, a slice thickness of 0.4 mm, and an inter-slice spacing of 0.4 mm, indicating a nominal isotropic voxel spacing of 0.4 mm. The datasets were imported into NemoFAB (Version 2025, Nemotec, Madrid, Spain), where automated anatomical segmentation and three-dimensional reconstruction were performed. For the finite element analysis, the maxillary bone was represented as separate cortical and trabecular volumes. Virtual segmental Le Fort I osteotomies were subsequently performed according to the planned surgical treatment, and the resulting anatomical models were exported as stereolithography (STL) files. The STL models were then imported into 3-matic Medical (Version 18.0, Materialise NV, Leuven, Belgium), where the patient-specific titanium fixation systems were designed.

### 2.2. Design of the Patient-Specific Fixation Systems

This study compared complete locking and non-locking patient-specific fixation configurations rather than isolating a single locking variable. Within each anatomical model, the paired configurations shared the same skeletal geometry, plate design and position, material assignments, loading conditions, and boundary conditions but differed simultaneously in screw-head and thread geometry and in the plate–screw contact formulation. Because these two factors were not varied independently, their individual contributions cannot be distinguished, and the within-model results were therefore interpreted as configuration-level responses rather than as an isolated effect of the locking mechanism.

Four patient-specific fixation configurations were developed and subsequently analysed by finite element analysis. Two plate geometries were investigated: a conventional design reproducing the standard clinical fixation configuration and a reduced, minimally invasive design. The reduced patient-specific plate design was developed to decrease implant volume, minimise soft-tissue coverage, and facilitate surgical handling while maintaining adequate biomechanical stability. It was specifically intended for selected segmental Le Fort I osteotomies in patients with skeletal Class II dentofacial deformities, in whom the anatomical characteristics and planned surgical movements allow a reduction in implant dimensions with the aim of maintaining adequate fixation stability. Consequently, this design was evaluated within its intended clinical indication and should not be considered a universal alternative to the standard patient-specific fixation system.

Each plate design was evaluated in both locking and non-locking fixation configurations, yielding four complete fixation configurations. The plates were customised to each patient’s anatomy and positioned along the principal maxillary load-bearing structures. In the standard design the plates engaged both the nasomaxillary and the zygomaticomaxillary buttresses; in the reduced design the framework was confined to the nasomaxillary region and included a transverse anterior component spanning the interdental osteotomies. Plate thickness varied from 0.9 to 1.0 mm in the bridging regions and from 1.3 to 2.0 mm around the fixation holes.

**Figure 1:**
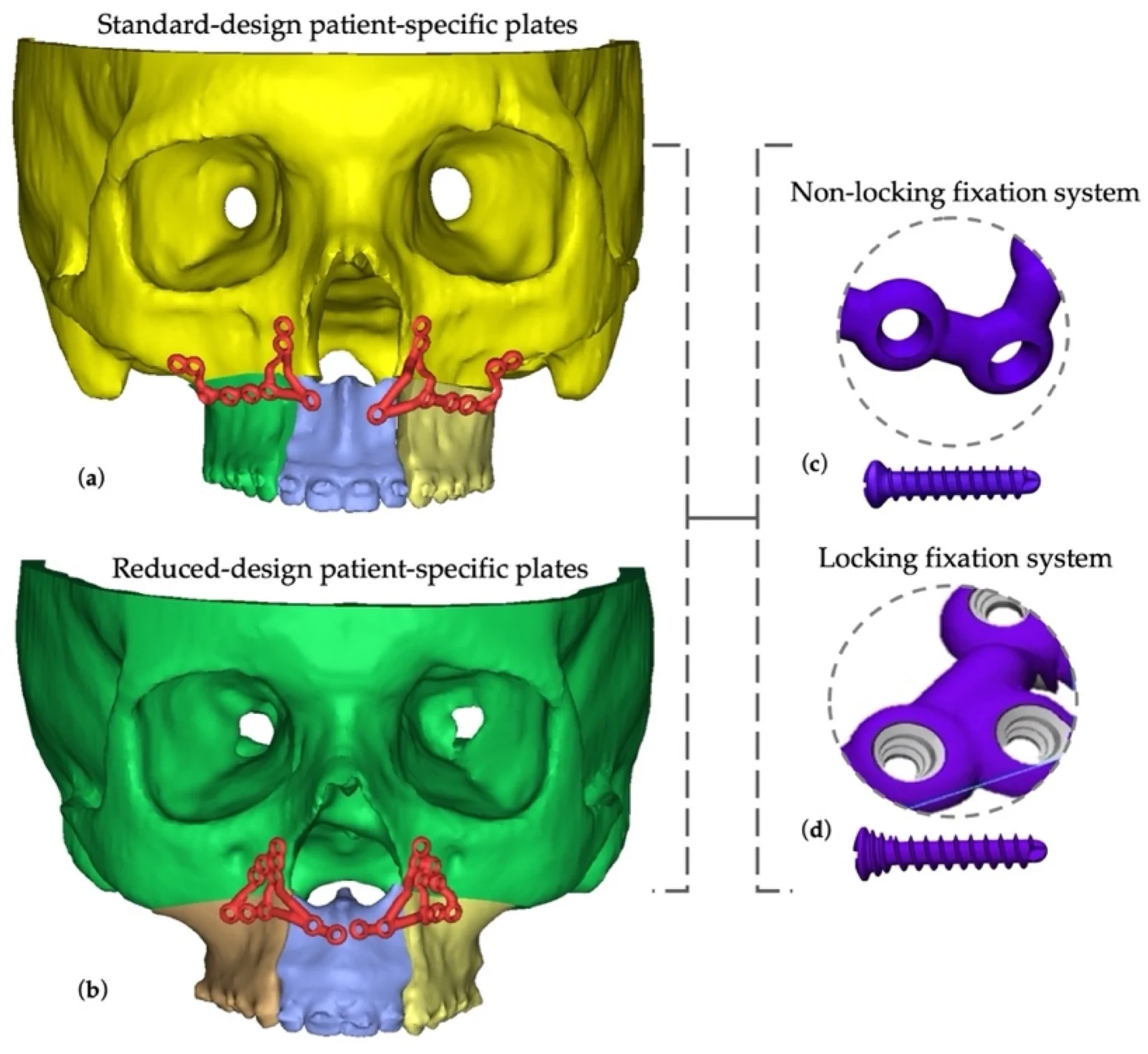
Frontal view of the three-dimensional reconstructions of the two patient-specific cranio-maxillofacial models and their corresponding titanium fixation systems for segmental Le Fort I osteotomy: (**a**) standard design applied to the skeletal Class III patient model, engaging both the nasomaxillary and zygomaticomaxillary buttresses; (**b**) reduced, minimally invasive design applied to the skeletal Class II patient model, confined to the nasomaxillary region; (**c**) the non-locking screw–plate interface, and (**d**) the locking screw–plate interface. The combination of the two plate designs with the two fixation mechanisms yielded the four analysed configurations: standard non-locking, standard locking, reduced non-locking, and reduced locking.

Two screw systems were represented as components of the complete locking and non-locking fixation configurations. The non-locking system used commercially available titanium miniscrews measuring 2.0 mm in diameter and 5 mm in length. The locking system used virtual models of custom-designed locking screws developed by our research team, featuring a 5 mm threaded shaft and a threaded head that engaged the corresponding threaded holes in the fixation plates (Figure 1).

### 2.3. Finite Element Model Generation

After completion of the design process, all anatomical structures and fixation components were exported as stereolithography (STL) files. The models underwent geometric smoothing using Geomagic Freeform (Version 2024, Hexagon AB, Stockholm, Sweden) and were subsequently converted into STEP (.stp) format. The resulting geometries were imported into HyperMesh (Altair Engineering Inc., Troy, MI, USA) for finite element preprocessing and mesh generation before being transferred to ANSYS Mechanical 2025 R2 (Ansys Inc., Canonsburg, PA, USA) for numerical analysis. All computational models were discretised using second-order tetrahedral elements.

### 2.4. Material Properties

The finite element models were assumed to be homogeneous, isotropic and linearly elastic, consistent with the approach adopted in comparable maxillofacial finite element studies [18,26,27,28]. Cortical bone was assigned a Young’s modulus of 14.8 GPa and a Poisson’s ratio of 0.35, and trabecular bone a Young’s modulus of 1.85 GPa and a Poisson’s ratio of 0.30. In the finite element models, the patient-specific fixation plates and screws were represented as Ti–6Al–4V alloy and were assigned a Young’s modulus of 110 GPa and a Poisson’s ratio of 0.30. These material parameters were adopted from previously published finite element data for Le Fort I osteotomy fixation [18].

### 2.5. Boundary Conditions and Loading

**Figure 2:**
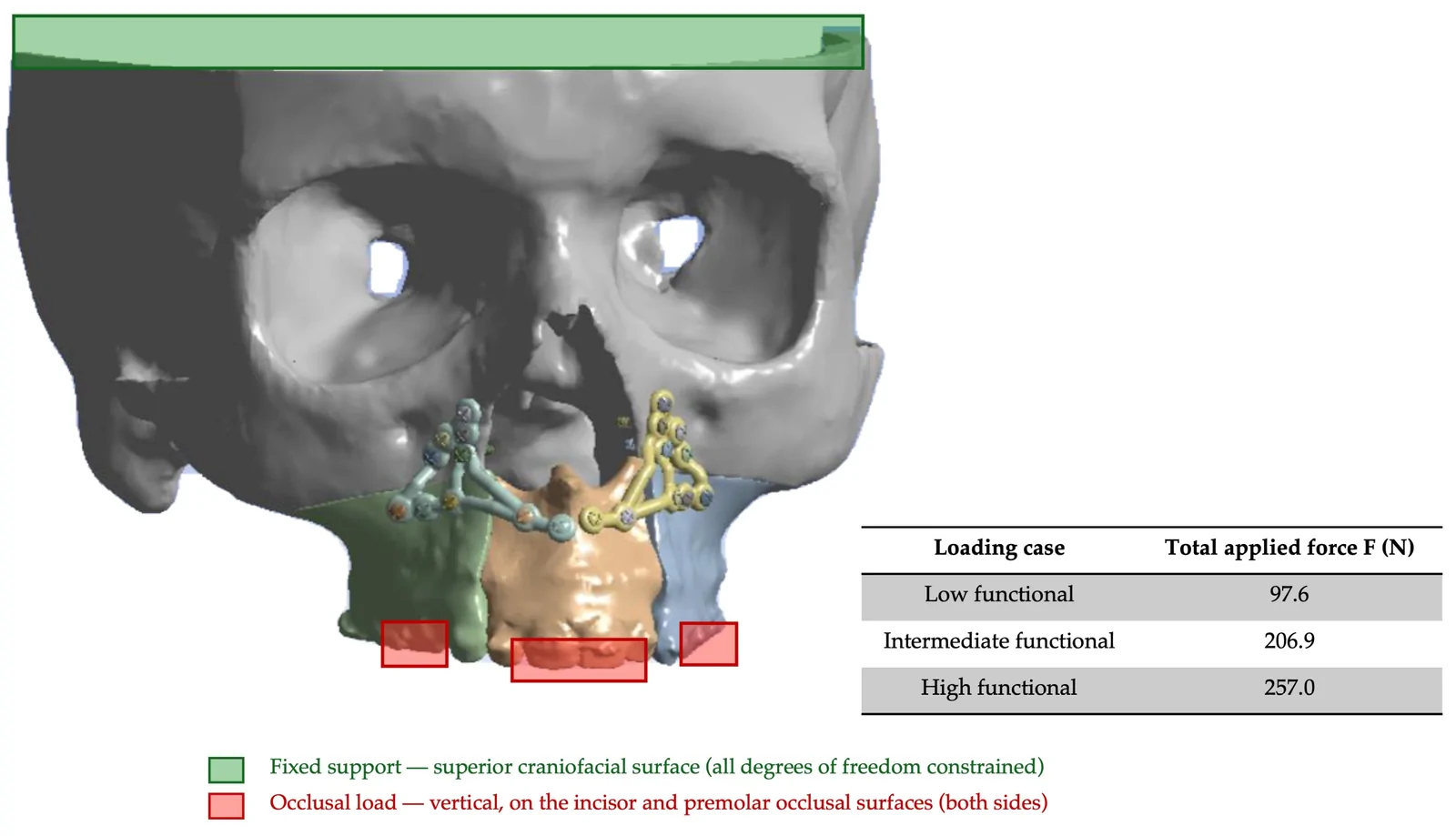
Frontal view of the patient-specific model showing the three segments produced by the segmental Le Fort I osteotomy and the patient-specific fixation system; the superior craniofacial surface, constrained in all directions, is shown in green, and the loaded occlusal surfaces are shown in red, with arrows indicating the direction of the applied vertical forces.

Three quasi-static loading conditions were evaluated using vertical forces of 97.6, 206.9, and 257.0 N. Based on previously reported changes in occlusal force following orthognathic surgery, these values were selected to represent low, intermediate, and high functional loading levels [18,29,30]. The superior surface of each craniofacial model was fully constrained in all directions to reproduce the anatomical continuity of the maxilla with the neurocranium and to eliminate rigid-body motion. The forces were applied bilaterally to the incisor and premolar regions. Within each patient-specific anatomical model, the loading locations, directions, and force distribution were kept identical for the locking and non-locking fixation configurations (Figure 2).

Contact interactions were defined at the bone–plate, plate–screw, screw–bone and osteotomy interfaces. Bonded contact was assigned at the bone–plate interface in all configurations as an idealisation of complete seating of the fixation plate against the cortical bone, with no relative sliding or separation. The same formulation was applied to all four configurations to maintain consistent interface conditions and avoid introducing bone–plate contact behaviour as an additional variable. At the plate–screw interface, bonded contact was assigned to the locking configurations to represent the angular-stable engagement of the threaded locking head in the threaded plate hole, whereas frictional contact with a coefficient of friction of 0.3 was assigned to the non-locking configurations with the intention of representing compression-dependent, non-rigid engagement [31]. The screw–bone interface was modelled as bonded in all configurations as an idealisation of complete mechanical engagement, with no relative sliding or separation. This formulation does not reproduce interface slip, screw loosening, or local interface failure. The osteotomy interfaces were modelled using a bonded contact formulation, which imposed displacement continuity and prevented separation and relative sliding between the adjacent bone segments.

For each fixation configuration and loading condition, the numerical outputs comprised the equivalent von Mises stress and total displacement in the maxillary bone, fixation plates, and screws.

Solver-reported maximum equivalent von Mises stresses were retained as descriptive outputs for transparency. Because isolated nodal maxima located at geometric or contact discontinuities may be mesh-dependent and non-convergent, they were not considered physically representative stress estimates or used to quantitatively rank the fixation configurations. Comparative interpretation focused primarily on component displacement patterns, while the stress distributions were used to identify stress-concentration regions. The mesh-convergence assessment was limited to maximum segmental displacement and representative non-singular plate stress. The solver-reported global stress maxima in the bone, plates, and screws were not included in this assessment.

### 2.6. Numerical Analysis and Output Evaluation

Static structural analyses were conducted using the Static Structural module of Ansys Mechanical 2025 R2 (Ansys Inc., Canonsburg, PA, USA). All simulations were performed on an HP Z840 dual-socket workstation (HP Inc., Palo Alto, CA, USA) equipped with two Intel Xeon E5-2699 v4 processors (Intel Corporation, Santa Clara, CA, USA; 22 physical cores and 44 threads per processor, 2.2 GHz base frequency, 3.6 GHz maximum turbo frequency, and 55 MB L3 cache), providing a total of 44 physical cores and 88 logical threads. The workstation also included 512 GB of DDR4-2400 ECC LRDIMM memory, an NVIDIA Quadro P6000 professional graphics card with 24 GB of GDDR5X memory (NVIDIA Corporation, Santa Clara, CA, USA) for graphical acceleration and interactive post-processing, and a 2 TB NVMe PCIe solid-state drive. The operating system was Microsoft Windows 10 Pro for Workstations (64-bit). Ansys Mechanical was configured to use the sparse direct solver in shared-memory parallel mode, with 32 physical cores allocated to each analysis.

For each component, total displacement was calculated as the Euclidean norm of the three displacement components, √(u_x^2^ + u_y^2^ + u_z^2^). The maximum total displacement was evaluated across all nodes belonging to each complete component. For every analysis, the solver automatically identified the node corresponding to the maximum value, using the same whole-component extraction procedure across all loading conditions and fixation configurations.

For a representative fixation configuration subjected to the intermediate functional load of 206.9 N, one complete static structural analysis required approximately 92 min of wall-clock time. This included approximately 78 min for contact detection, equilibrium iterations, and solution using the sparse direct solver, followed by approximately 14 min for result extraction and post-processing.

## 3. Results

### 3.1. Mesh Convergence

**Figure 3:**
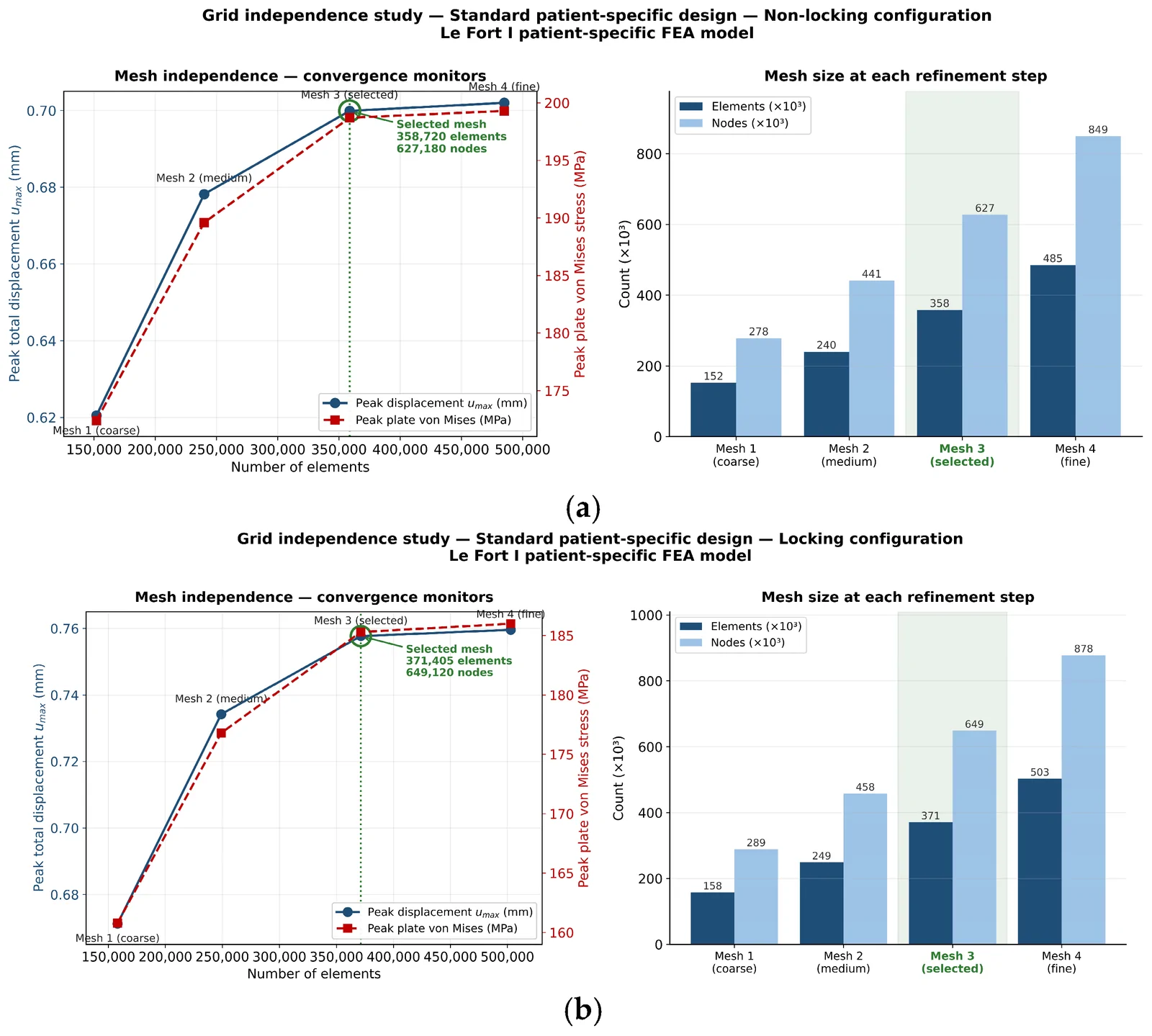

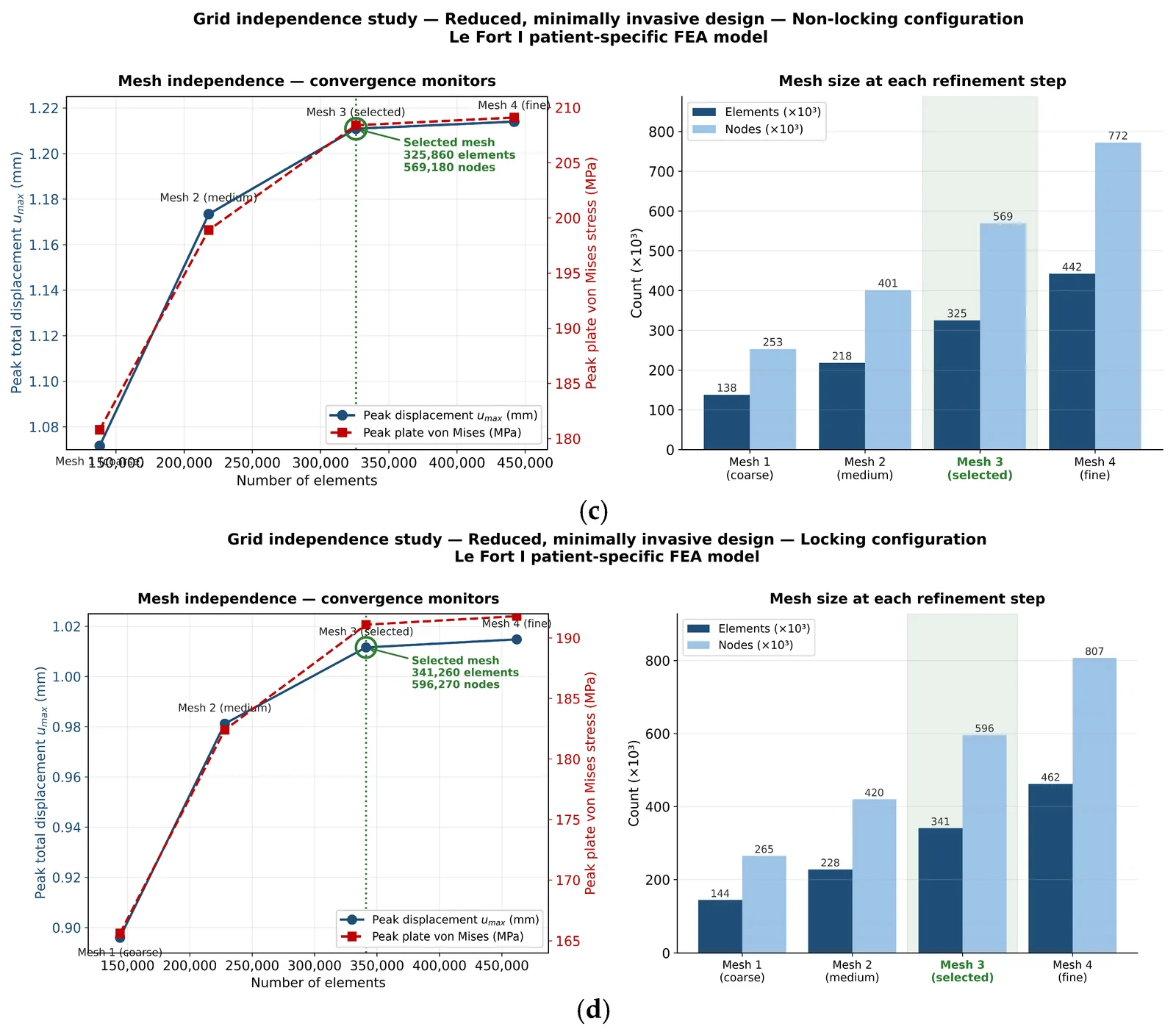
Mesh-convergence assessment for the four patient-specific fixation configurations: (**a**) standard non-locking; (**b**) standard locking; (**c**) reduced non-locking; and (**d**) reduced locking. The left panels show maximum segmental displacement and representative non-singular plate stress against the number of elements, whereas the right panels show the element and node counts at each refinement level. The selected meshes are highlighted in green.

Mesh convergence was assessed separately for the four fixation configurations using four successively refined meshes with global element sizes of 2.00, 1.50, 1.00, and 0.75 mm. Under the intermediate functional load of 206.9 N, convergence was evaluated using maximum segmental displacement and representative plate equivalent von Mises stress measured away from singular regions. The predefined convergence criteria were relative changes below 1% for displacement and 2% for plate stress. Between the selected and finest meshes, displacement changes ranged from 0.25% to 0.30%, while stress changes remained below 2%, confirming adequate mesh convergence. The complete mesh characteristics and convergence results are presented in Figure 3 and Table 1.

### 3.2. Standard Patient-Specific Fixation Model: Locking Versus Non-Locking

**Figure 4:**
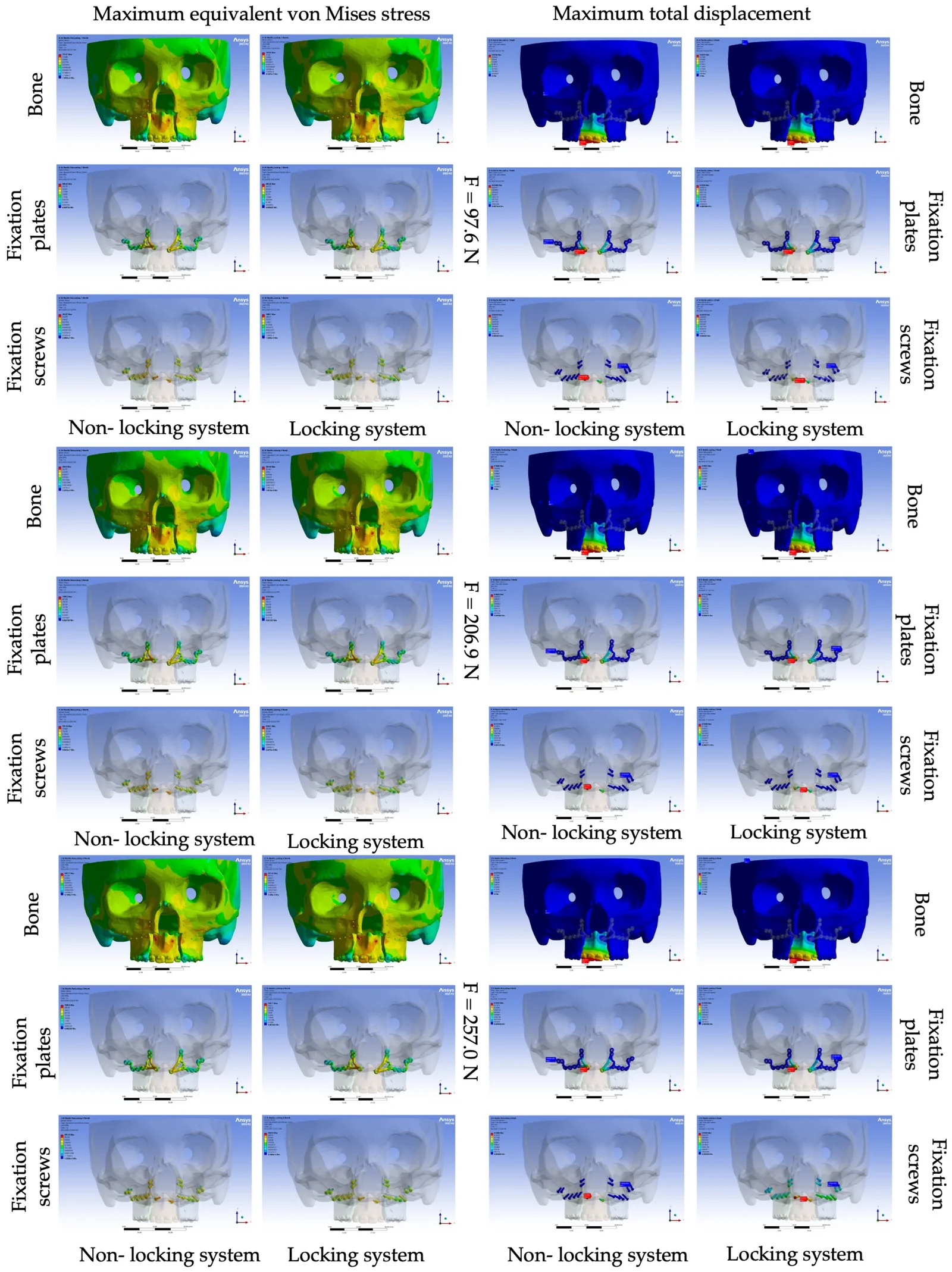
Standard patient-specific design: equivalent von Mises stress (MPa, (**left block**)) and total displacement (mm, (**right block**)) in the maxillary bone, fixation plates and fixation screws, for the non-locking and locking configurations at 97.6, 206.9 and 257.0 N. In the total displacement maps, the red and blue arrows indicate the locations of maximum and minimum displacement, respectively.

In the standard patient-specific fixation model, the maximum equivalent von Mises stress and the maximum total displacement increased approximately proportionally with the applied occlusal load in both fixation configurations. Under the highest loading condition (257.0 N), the locking configuration exhibited greater bone displacement (0.944 versus 0.872 mm), plate displacement (0.145 versus 0.134 mm), and screw displacement (0.161 versus 0.143 mm) than the corresponding non-locking configuration. The solver also returned local maximum screw stresses of 3922.80 MPa in the locking configuration and 879.15 MPa in the non-locking configuration (Table 2 and Figure 4). Because these isolated maxima occurred at contact-sensitive regions and the plate–screw contact formulations differed between the configurations, the values are reported as raw solver outputs and were not used to quantify relative screw loading or infer yielding.

### 3.3. Reduced Patient-Specific Fixation Model: Locking Versus Non-Locking

**Figure 5:**
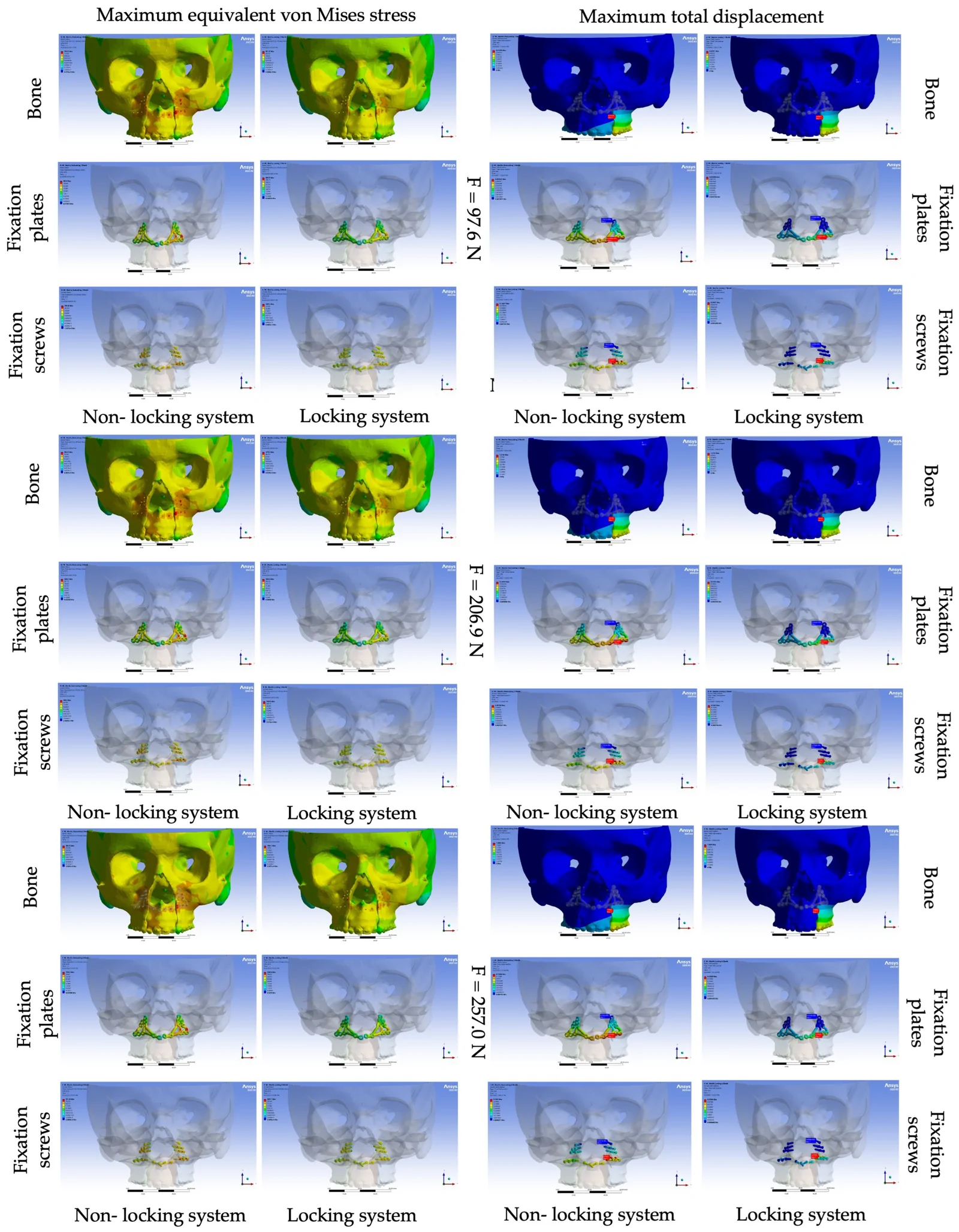
Reduced, minimally invasive patient-specific design: equivalent von Mises stress (MPa, (**left block**)) and total displacement (mm, (**right block**)) in the maxillary bone, fixation plates and fixation screws, for the non-locking and locking configurations at 97.6, 206.9 and 257.0 N. In the total displacement maps, the red and blue arrows indicate the locations of maximum and minimum displacement, respectively.

A similar load-dependent trend was observed in the reduced patient-specific fixation model, with maximum equivalent von Mises stress and maximum total displacement increasing progressively in both fixation configurations. At 257.0 N, the locking configuration exhibited lower computed displacements than the corresponding non-locking configuration, with reductions of approximately 16.4% in bone displacement, 19.2% in plate displacement, and 16.3% in screw displacement; construct stiffness itself was not calculated. At the same loading level, the solver-reported maximum stresses in the locking configuration were 1584.10 MPa in the bone, 2340.90 MPa in the plates, and 3927.10 MPa in the screws, compared with 358.13, 1324.20, and 671.26 MPa, respectively, in the non-locking configuration (Table 3 and Figure 5). These values are reported descriptively because they represent isolated local maxima whose spatial extent was not quantified. Accordingly, the contact-sensitive screw peaks were not used to quantitatively compare overall screw loading between the configurations. Similarly, the bone maximum of 1584.10 MPa was not considered a spatially averaged estimate of physiological bone stress or a prediction of local bone failure, particularly because the linear-elastic bone model did not incorporate yielding, damage, or post-yield stress redistribution.

### 3.4. Descriptive Overview of Model-Specific Biomechanical Outcomes

**Figure 6:**
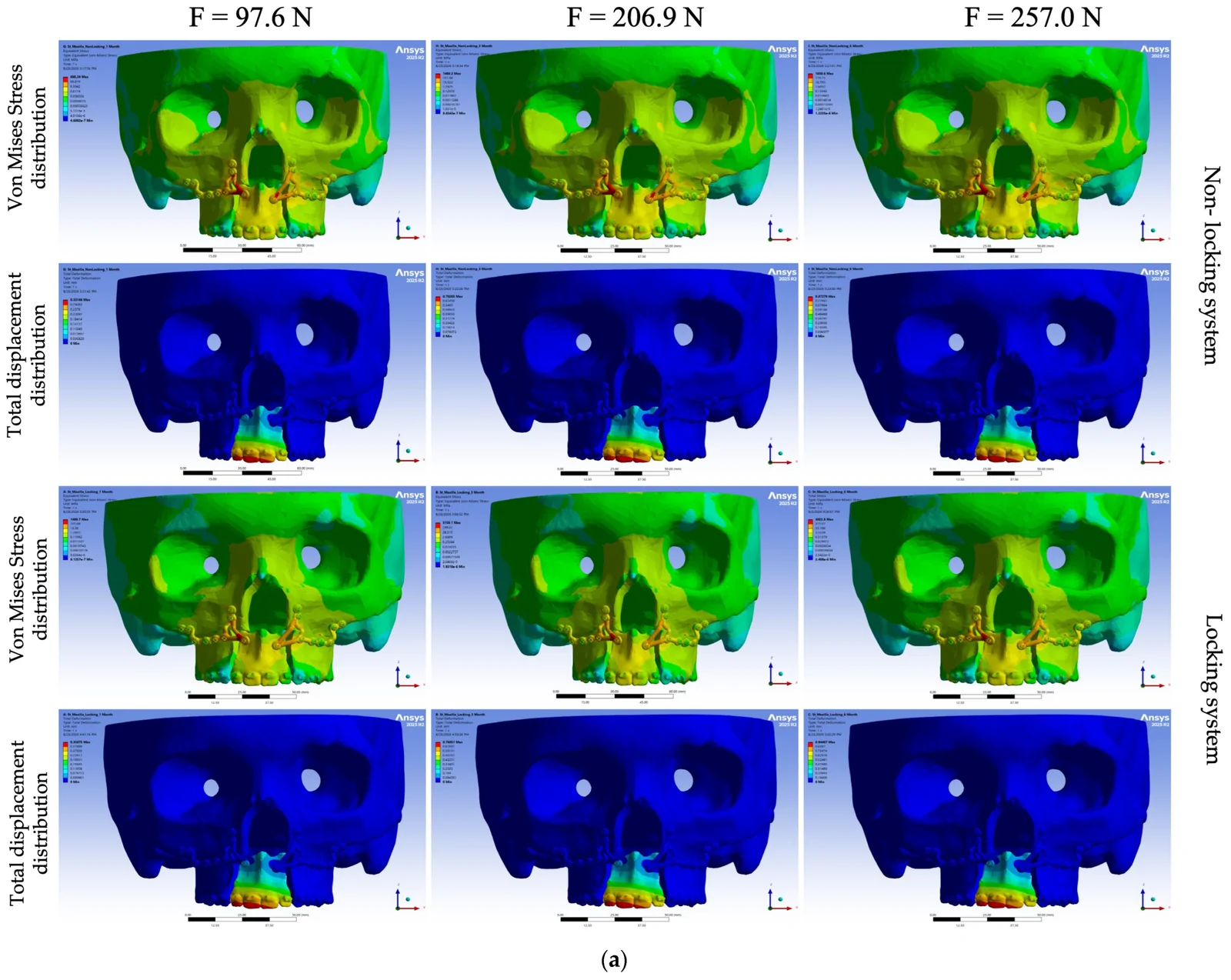

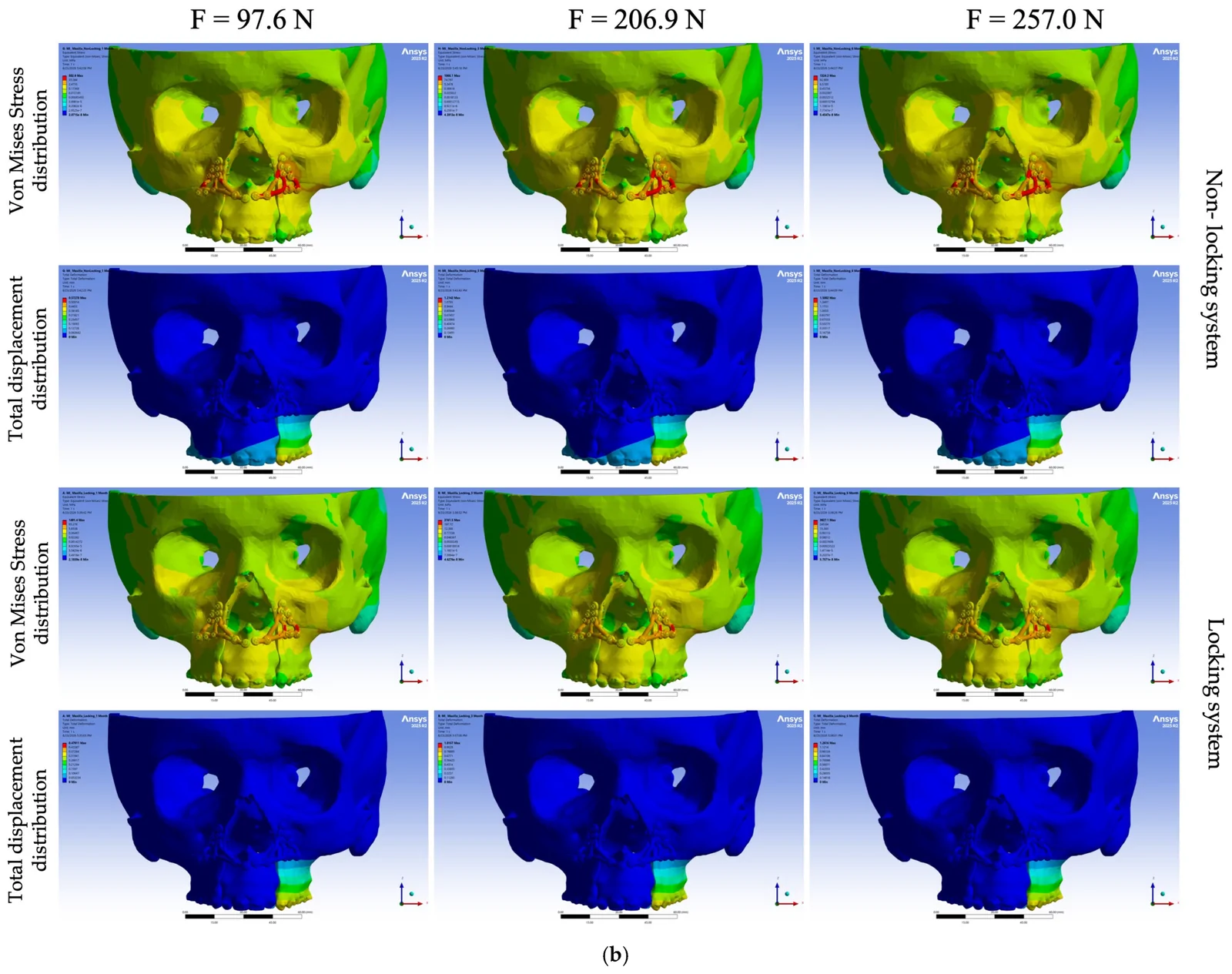
Whole-model distributions of equivalent von Mises stress and total displacement under loads of 97.6, 206.9, and 257.0 N for the non-locking and locking configurations: (**a**) standard design and (**b**) reduced design.

In both patient-specific models, the solver-reported global maximum equivalent von Mises stress and maximum total displacement increased proportionally with the applied occlusal load. The relative displacement differences between the locking and non-locking configurations remained consistent across the three loading levels but differed between the two anatomical models. The global stress maxima occurred in the fixation screws of the locking configurations and in the fixation plates of the non-locking configurations. Although representative non-singular plate stress was used as a convergence monitor, the solver-reported global stress maxima were not included in the mesh-convergence assessment, and no region-averaged or percentile-based stress measures were calculated for these maxima. Therefore, they were not used to quantitatively rank the fixation configurations. Elevated stresses were localised around the osteotomy and fixation regions, whereas the greatest displacement occurred within the mobilised dentoalveolar segments (Figure 6).

## 4. Discussion

The present finite element study compared four complete patient-specific fixation configurations for segmental Le Fort I osteotomy. Within the standard model, the locking configuration exhibited slightly greater component displacement than the corresponding non-locking configuration, whereas within the reduced model, it exhibited lower bone, plate, and screw displacement. Because the paired configurations differed simultaneously in screw geometry and plate–screw contact formulation, the observed differences cannot be attributed solely to the locking mechanism. Furthermore, as no controlled parametric analysis was performed, the present results cannot determine the independent contributions or interactions of fixation characteristics, plate geometry, screw distribution, contact assumptions, and patient-specific anatomy. They should therefore be interpreted as model-specific responses under the prescribed modelling assumptions.

The approximately proportional increase in stress and displacement with increasing load was consistent with the linear-elastic assumptions of the analysis. Given that material properties, osteotomy stiffness, contact definitions, and boundary conditions were held constant, the three loading scenarios represent scaled responses within each computational model and should not be interpreted as simulations of biological healing processes or postoperative recovery conditions. Previous finite element studies of Le Fort I fixation have shown that plate position, plate number, screw distribution, and the relationship between the fixation system and the vertical maxillary buttresses can influence load transfer and segment stability [18,19,26]. Clinical studies comparing two- and four-plate fixation constructs have also highlighted the potential importance of fixation point distribution in postoperative skeletal stability [13,14].

In the reduced patient-specific model, maximum bone displacement reached 1.508 mm in the non-locking configuration and 1.261 mm in the locking configuration at 257.0 N. These values represent maximum computational displacement at specific locations and should not be interpreted as uniform mobility of the osteotomised maxilla or as direct measures of intersegmental micromotion.

The solver-reported local maximum screw stresses of approximately 3923–3927 MPa in the locking configurations exceed the reported yield strength of additively manufactured Ti–6Al–4V. Jamshidi et al. [20] reported 0.2% proof stresses of 760.9 ± 22.3 MPa and 820.1 ± 16.5 MPa for horizontally and vertically oriented as-built selective-laser-melted specimens, respectively, increasing to approximately 865 MPa after hot isostatic pressing and polishing. However, the calculated maxima should not be interpreted as predictions of screw yielding or failure because the linear-elastic model does not simulate the onset of plastic deformation or the subsequent redistribution of stress. These values likely reflect two non-exclusive factors. First, the locking screw head engages the threaded plate hole at a sharp, re-entrant geometric transition, where the highest nodal stress may continue to increase with mesh refinement rather than converge to a finite physical value [32]. Second, the bonded plate–screw contact used to represent locking engagement constitutes a more constrained interface than the frictional contact assigned to the non-locking configurations, and contact formulation can substantially influence peak stresses in otherwise comparable plate–screw constructs [31]. Comparable mechanical studies of CAD/CAM fixation systems have similarly reported that changes in screw–plate interaction can alter local mechanical behaviour without necessarily producing equivalent changes in global construct stiffness [33]. Because screw geometry and plate–screw contact formulation varied simultaneously, and because the isolated screw-stress maxima were not included in the mesh-convergence assessment, these values cannot be used to compare overall screw loading quantitatively. Although representative non-singular plate stress was evaluated for convergence, no region-averaged or percentile-based screw stress measure was calculated.

Hardware-related complications, including plate detachment and fracture, have been documented in long-term clinical cohorts following orthognathic fixation, although they remain uncommon [34]. Evidence from the broader maxillofacial literature indicates that titanium fixation systems are removed more frequently because of infection than because of mechanical failure [35]. The clinical significance of the high computed screw stresses therefore remains uncertain and, if relevant at all, should be regarded as a contributing biomechanical factor rather than an independent predictor of hardware failure.

A similarly prominent caution applies to the solver-reported maximum bone stress of 1584.1 MPa in the reduced locking configuration. This value is approximately one order of magnitude greater than commonly reported cortical bone strength values [36] and, if interpreted literally, would imply immediate local tissue failure. Such an interpretation is not supported by the present modelling approach. Cortical and trabecular bone were represented as homogeneous, isotropic, and linearly elastic materials; consequently, the model did not reproduce yielding, microdamage, stiffness degradation, anisotropy, density-dependent heterogeneity, or the redistribution of stress following local tissue damage. Under these assumptions, the solver permits stress to increase beyond the physical strength of bone without modifying the local material response. Moreover, the reported value represents an isolated maximum whose spatial extent was not quantified. This global bone-stress maximum was not included in the mesh-convergence assessment, and no region-averaged or percentile-based bone stress measure was calculated. Equivalent von Mises stress also does not fully represent the tension–compression asymmetry and direction-dependent failure behaviour of cortical bone. Therefore, the value of 1584.1 MPa should not be interpreted as a physiological tissue stress, a safety-factor estimate, or direct evidence that the reduced locking configuration is clinically impractical. Nevertheless, it identifies the surrounding fixation region as a numerically critical area requiring further evaluation.

The findings have several implications for patient-specific implant design. The reduced, minimally invasive system may offer advantages in implant volume, soft-tissue coverage and surgical access, and its use should remain indication-specific. Because the two designs were evaluated in different anatomies, however, these results cannot establish whether confining the framework to the nasomaxillary region compromises anchorage; answering that would require both designs in the same model.

For additively manufactured Ti–6Al–4V fixation systems, the fatigue limit of the as-built alloy is reduced relative to its static strength by surface roughness and near-surface porosity, whereas post-processing that reduces roughness improves both ductility and fatigue performance [20]. A persistent stress-concentration region at a threaded interface is therefore of greater concern under cyclic masticatory loading than a static comparison with yield strength would suggest. Accordingly, increasing the cross-sectional area at high-stress transitions, smoothing abrupt changes in curvature, optimising inter-hole spacing, redistributing fixation points, and selectively reinforcing the locking interfaces are design modifications that warrant evaluation in future parametric studies as potential means of improving load sharing while preserving the minimally invasive character of the reduced device.

### Study Limitations

Several limitations inherent to the computational methodology should be acknowledged. The models did not incorporate time-dependent bone healing, callus formation, periodontal ligament behaviour, muscle forces, or postoperative changes in occlusion. Bone and Ti–6Al–4V were represented using simplified linear-elastic material models; therefore, yielding, damage, plastic deformation, and the subsequent redistribution of stress were not simulated. The dentition was used as the surface through which the occlusal load was applied, but its material assignment and attachment conditions were not characterised separately. These simplifications may affect both the local stress fields and the computed displacement patterns. Loading was static, vertical, and unidirectional, whereas mastication involves repetitive, multidirectional, and patient-specific forces. No fatigue analysis was performed, and manufacturing tolerances, surface roughness, residual stresses, or screw preload were not represented.

The bonded bone–plate, screw–bone, and osteotomy contact formulations represent additional modelling simplifications. By preventing relative sliding and separation, these formulations do not reproduce clinically possible bone–plate micromotion, screw–bone slip, screw loosening, local interface failure, or relative intersegmental motion across the osteotomy. They may artificially increase construct stiffness and alter the predicted load transfer and stress distributions in the bone, plates, and screws. Because no contact-sensitivity analyses were performed, the magnitude and direction of these effects could not be quantified.

No controlled sensitivity analysis independently varied screw geometry and plate–screw contact formulation; consequently, their individual contributions could not be determined. Sensitivity or uncertainty analyses were also not performed for the material properties or the location of the constrained surface. Maximum total displacement was reported for entire components and did not quantify relative intersegmental motion across the osteotomy sites. Equivalent von Mises stress was used as a consistent descriptive metric, although principal stresses and strains would provide complementary information regarding the tension–compression-dependent behaviour of bone.

Neither patient-specific model was experimentally validated. Accordingly, the results represent comparative computational findings and cannot be directly translated into predictions of implant failure, local bone damage, postoperative skeletal relapse, or the clinical superiority of a particular fixation configuration. Skeletal relapse is influenced by the direction and magnitude of maxillary movement, fixation strategy, healing-related remodelling, muscular loading, postoperative occlusion, and patient-specific biological factors [11,12,15]. Moreover, a recent clinical cohort found no significant association between conventional and minimally invasive patient-specific plate designs and postoperative skeletal instability [37]. This further emphasises that numerical differences observed under simplified static loading should not be interpreted as evidence of corresponding differences in clinical stability.

Clinical selection should instead consider the complete patient-specific fixation configuration, including plate geometry, screw number and distribution, plate–screw engagement, the planned maxillary movement, and patient-specific anatomy. In the configurations analysed, displacement differences were model-dependent, whereas the isolated local stress maxima were contact- and mesh-sensitive; consequently, greater numerical constraint should not automatically be interpreted as superior clinical performance. Similarly, a reduced, minimally invasive design may be considered when adequate anchorage and continuous load-transfer pathways can be maintained, but it should not be selected solely on the basis of reduced implant volume. The principal clinical implication is that patient-specific fixation should balance construct stability with distributed load sharing while minimising abrupt geometric and contact transitions at critical fixation interfaces.

Future studies should include a controlled factorial sensitivity analysis comparing identical screw geometries under alternative contact formulations and different screw geometries under identical contact conditions, while maintaining the same anatomy, plate geometry, mesh, loading, and boundary conditions. Bone–plate and screw–bone contacts should also be evaluated using frictional or nonlinear formulations incorporating clinically relevant friction coefficients, screw preload, interface slip, loosening, and local interface failure. The present mesh-convergence assessment should be extended to component-specific regional stress measures, particularly in the bone and screws, including stresses evaluated at a defined distance from singular regions, area- or volume-averaged stresses, and percentile-based measures. Principal stress, strain, and failure criteria should also be considered, particularly for bone. Further improvements should include elastoplastic modelling of Ti–6Al–4V, anisotropic nonlinear or damage-based bone behaviour, healing-dependent changes in osteotomy stiffness, and multiple loading directions. Finally, patient-specific fixation constructs should be tested using standardised printed bone analogues under loading and boundary conditions corresponding to those used in the finite element models. Experimentally measured construct displacement, intersegmental motion, strain distribution, and failure behaviour should then be compared with the numerical predictions using previously validated bone-analogue testing approaches [38], followed by prospective clinical validation.

## 5. Conclusions

Within the limitations of this finite element study, the locking and non-locking configurations evaluated within the two patient-specific maxillary models exhibited distinct biomechanical responses across the three functional loading levels. The locking configuration was associated with greater component displacement in the standard model but lower displacement in the reduced model, demonstrating that its biomechanical response was model-dependent. Stress distributions identified the fixation interfaces as contact- and geometry-sensitive stress-concentration regions; however, the isolated maximum stresses in the bone and fixation components were not regarded as physically representative values and were not used to rank the configurations or predict material failure. From a clinical perspective, these findings support configuration-level optimisation aimed at balancing mechanical stability with distributed load transfer; they do not establish an independent biomechanical advantage of the locking mechanism.

## Figures and Tables

**Table 1:** Mesh-refinement parameters and convergence results for the four patient-specific fixation configurations under the intermediate functional load of 206.9 N.

Mesh Level	Element Size (mm)	Elements	Nodes	u_max (mm)	Δu vs. Prev. (%)	σ_plate Non-Sing. (MPa)	Δσ vs. Prev. (%)
Standard—non-locking							
Mesh 1 (coarse)	2.00	152,400	278,600	0.620	—	172.4	—
Mesh 2 (medium)	1.50	240,150	441,300	0.678	+9.35%	189.6	+9.98%
Mesh 3 (selected)	1.00	358,720	627,180	0.702	+3.54%	198.7	+4.80%
Mesh 4 (fine)	0.75	485,930	849,540	0.704	+0.28%	199.4	+0.35%
Standard—locking							
Mesh 1 (coarse)	2.00	158,930	289,270	0.671	—	160.8	—
Mesh 2 (medium)	1.50	249,470	458,010	0.734	+9.39%	176.9	+10.01%
Mesh 3 (selected)	1.00	371,405	649,120	0.760	+3.54%	185.4	+4.81%
Mesh 4 (fine)	0.75	503,220	878,700	0.762	+0.26%	186.1	+0.38%
Reduced—non-locking							
Mesh 1 (coarse)	2.00	138,240	253,120	1.072	—	180.9	—
Mesh 2 (medium)	1.50	218,110	401,880	1.173	+9.42%	199.0	+10.01%
Mesh 3 (selected)	1.00	325,860	569,180	1.214	+3.50%	208.5	+4.77%
Mesh 4 (fine)	0.75	442,100	772,240	1.217	+0.25%	209.2	+0.34%
Reduced—locking							
Mesh 1 (coarse)	2.00	144,750	265,340	0.896	—	165.7	—
Mesh 2 (medium)	1.50	228,280	420,860	0.981	+9.49%	182.4	+10.08%
Mesh 3 (selected)	1.00	341,260	596,270	1.015	+3.47%	191.1	+4.77%
Mesh 4 (fine)	0.75	462,470	807,540	1.018	+0.30%	191.8	+0.37%

The selected mesh for each configuration is shown in italics. u_max—the maximum total displacement of the mobilised maxillary segment; Δu—the relative change from the preceding mesh level; σplate non-sing.—the equivalent von Mises stress evaluated within a predefined fixation-plate region located away from geometric and contact singularities; Δσ—the relative change from the preceding mesh level.

**Table 2:** Maximum equivalent von Mises stress (MPa) and maximum total displacement (mm) in the maxillary bone, patient-specific fixation plates, and fixation screws for the standard configuration under three functional loading conditions (97.6, 206.9, and 257.0 N).

Component	Occlusal Load	Maximum Equivalent von Mises Stress (MPa)		Maximum Total Displacement (mm)	
		Standard (Non-Locking)	Standard (Locking)	Standard (Non-Locking)	Standard (Locking)
Bone	97.6 N	117.41	122.03	0.331	0.358
	206.9 N	248.90	258.68	0.702	0.760
	257.0 N	309.17	321.32	0.872	0.944
Fixation Plates	97.6 N	698.24	646.25	0.051	0.055
	206.9 N	1480.20	1370.00	0.108	0.117
	257.0 N	1838.60	1701.70	0.134	0.145
Fixation Screws	97.6 N	333.87	1489.70	0.054	0.061
	206.9 N	707.76	3158.10	0.115	0.129
	257.0 N	879.15	3922.80	0.143	0.161

The reported stress values represent raw solver maxima retained for transparency. They were not treated as physically converged stress estimates or used to quantitatively rank the fixation configurations or predict material failure.

**Table 3:** Maximum equivalent von Mises stress (MPa) and maximum total displacement (mm) in the maxillary bone, patient-specific fixation plates, and fixation screws for reduced configuration under three functional loading conditions (97.6, 206.9, and 257.0 N).

Component	Occlusal Load	Maximum Equivalent von Mises Stress (MPa)		Maximum Total Displacement (mm)	
		Reduced (Non-Locking)	Reduced (Locking)	Reduced (Non-Locking)	Reduced (Locking)
Bone	97.6 N	136.01	601.57	0.572	0.479
	206.9 N	288.31	1275.30	1.214	1.015
	257.0 N	358.13	1584.10	1.508	1.261
Fixation Plates	97.6 N	502.90	889.01	0.065	0.052
	206.9 N	1066.10	1884.60	0.139	0.112
	257.0 N	1324.20	2340.90	0.172	0.139
Fixation Screws	97.6 N	254.92	1491.40	0.126	0.105
	206.9 N	540.40	3161.50	0.267	0.224
	257.0 N	671.26	3927.10	0.332	0.278

The reported stress values represent raw solver maxima retained for transparency. They were not treated as physically converged stress estimates or used to quantitatively rank the fixation configurations or predict material failure.

## Data Availability

The original contributions presented in this study are included in the article. Further inquiries can be directed to the corresponding author.

## Abbreviations

The following abbreviations are used in this manuscript:

FEA	Finite Element Analysis
CAD/CAM	Computer-Aided Design/Computer-Aided Manufacturing
CBCT	Cone-Beam Computed Tomography
STL	Stereolithography
STEP	Standard for the Exchange of Product model data
3D	Three-dimensional
